# Coping strategies of school-going adolescents during the COVID-19 pandemic in the climate vulnerable Manafwa watershed, Uganda

**DOI:** 10.1186/s40359-024-01760-3

**Published:** 2024-05-29

**Authors:** Charles Batte, Shivan Nuwasiima, Andrew Weil Semulimi, Pamela Okwir Apio, Ronald Kasoma Mutebi, Martin Menya Mwesigwa, Nelson Twinamasiko, Trishul Siddharthan, John Mukisa, David Mukunya, Joan Abaatyo, Joyce Sserunjogi Nalugya

**Affiliations:** 1 Makerere Lung Institute, College of Health Sciences, Makerere University, Kampala, Uganda; 2 Climate and Health Unit, Tree Adoption, Kampala, Uganda; 3 Miller School of Medicine, University of Miami, Miami, USA; 4 Faculty of Medicine, Mbarara University of Science and Technology, Mbarara, Uganda; 5 Department of Medicine, Nakaseke General Hospital, Nakaseke, Uganda; 6 School of Biomedical Sciences, College of Health Sciences, Makerere University, Kampala, Uganda; 7 Faculty of Health Sciences, Busitema University, Mbale, Uganda; 8 Department of Psychiatry, Directorate of Medicine, Mulago National Referral Hospital, Kampala, Uganda

**Keywords:** Coping strategies, School-going adolescents, COVID-19 pandemic, Mental health, Uganda

## Abstract

**Background:**

The COVID-19 pandemic disrupted daily life, economies, and health, prompting strict government measures, including nationwide lockdowns and school closures in Uganda, resulting in significant academic setbacks for adolescents. The coping strategies employed by school-going adolescents in Uganda amidst the COVID-19 pandemic remain inadequately understood. This study aimed to assess the coping strategies adopted by school-going adolescents (early, middle and late adolescents) in the Manafwa watershed, recognized as one of Uganda’s most vulnerable regions, during the COVID-19 pandemic.

**Methods:**

A cross sectional study design was conducted from I5th May, 2023 to 30th June, 2023 on 762 school going adolescents from government-aided secondary schools within the Manafwa watershed area. The adolescent version of the KidCope tool was used to evaluate adolescents’ coping strategies. Factor analysis identified correlations among adolescents’ coping strategies. Independent Samples t-Test and One-Way Variance of Analysis (ANOVA) was used for comparing the mean score differences of the coping strategies among the gender and adolescents’ stages respectively.

**Results:**

Majority (*n* = 141, 36.2%) of the participants employed adaptive coping followed by negative-emotion coping (*n* = 127, 32.6%) and avoidant coping (*n* = 122, 31.3%). Females employed statistically higher resignation as a coping strategy compared to males, (mean of 1.2 vs. 1.0, respectively; *P* = 0.026). A higher proportion (*n* = 88, 69.3%) of middle age adolescents employed negative-emotion regulation (P-value = 0.040). However, those in early adolescence significantly utilized distraction as a coping strategy more than those in middle adolescence (mean difference = 0.36, *p* = 0.013).

**Conclusion:**

During the pandemic, majority of school-going adolescents employed adaptive coping mechanisms, including positive emotional regulation and social support. However, compared to males, females employed resignation more frequently. Moreover, middle-aged adolescents had a greater propensity for negative emotion copying. Findings from this study contribute valuable information for the development of targeted interventions and support mechanisms for adolescents facing unprecedented challenges.

## Introduction

Coronavirus disease (COVID-19) disrupted daily activities, economic well-being, and the health of individuals [1, 2]. More than 624 million people were diagnosed with COVID-19 while more than 6 million succumbed to it globally [3]. In Africa, more than 12 million people were confirmed to have COVID-19 with over 257,000 deaths [4]. Compared to other African nations like Kenya and South Africa, Uganda recorded less cases of COVID-19, with 3,632 deaths [5]. This was likely attributed to the proactive and strict nature of the government’s response to COVID-19. In order to control the spread of this highly contagious disease, countries worldwide implemented various levels of lockdowns and restrictions to curb transmission, with measures ranging from stay-at-home orders to border closures. Strict countrywide lockdowns as one of the measures instituted by the governments included the closure of communal places such as schools [6].

Despite school closures due to the COVID-19 pandemic, governments utilized various methods to uphold education, crucial for national development. Distance learning, including online and broadcast education via television and radio, became essential alternatives. Developed nations transitioned smoothly to online courses and exams, leveraging diverse applications and social networks, reaching 90% online delivery [7]. In contrast, only 23% of Sub-Saharan African developing countries provided broadcast and online learning due to poor internet connectivity, limited access to radios, TVs, and telephones among the poorest families [8, 9] and lack of enough technological skills among the teachers and students [10]. In Uganda, distance education favored urban students over rural ones like those in Manafwa, exacerbating pandemic effects on adolescent learners. Resource disparities and rural adolescents’ engagement in domestic activities also hindered their learning, compounding pandemic challenges for school-goers.

Regrettably, the closure of all institutions of learning by the Ugandan government cast the future of over 15 million learners in doubt [11]. Schools in Uganda were closed for nearly two years and school-going adolescents lost an enormous amount of study days [12]. The immediate effects of this on school-going adolescents have been devastating [13]. For instance, about 30% of students were not able to return to school while there was a surge in teenage pregnancies which rose by more than 20% during the first lockdown and this number was estimated to be much higher since the outbreak of COVID-19 [11, 14]. The school-going adolescents’ ability to advance to the next level of education was stunted, posing numerous challenges in young people’s lives [15]. When faced with such difficult circumstances, adolescents utilize certain coping strategies as regulatory processes aimed at mitigating the adverse emotional impacts of stressful situations [16–20].

Scholars have identified various coping strategies [19], which are typically classified within either the “approach-or-avoidance” model [21, 22], or the emotion- or problem-focused coping model. Problem-focused coping involves practical actions like problem-solving and seeking support to directly address or resolve the stressor [23, 24]. This approach is often linked with positive outcomes such as improved academic performance [25]. Studies have suggested that during the COVID-19 quarantine, adaptive problem-focused coping strategies contributed to greater psychological well-being [25, 26]. On the other hand, emotion-focused coping involves processing and expressing emotions triggered by the stressor [27]. It may include adaptive strategies like reappraisal or relaxation techniques [24, 28], but can also involve maladaptive approaches such as wishful thinking or avoidance or attempting to relax using breathing techniques [23]. Notably, research indicates that employing negative emotion-focused coping during the COVID-19 quarantine is associated with poorer psychological health, such as increased anxiety and depression [26]. Generally, research suggests that adaptive coping strategies are associated with positive outcomes [29].

The coping strategies employed by school-going adolescents in Uganda amid the COVID-19 pandemic remain inadequately understood and in order to provide supportive mechanisms to these adolescents so as to avoid negative impacts of future calamities, we need to understand the coping strategies employed by these adolescents. This study aimed to assess the coping strategies adopted by school-going adolescents, during the COVID-19 pandemic, in the Manafwa watershed, recognized as one of Uganda’s most vulnerable regions, due to frequent occurrence of landslides [30]. The findings from this research will provide valuable insights essential for developing targeted crisis response plans for future pandemics and related disasters.

## Methods

### Study design and setting

We conducted a cross-sectional study in the Manafwa watershed from I5th May, 2023 to 30th June, 2023. The Manafwa watershed is one of the bigger watersheds (502km^2^) on the slopes of Mountain Elgon in Eastern Uganda [31]. It constitutes of three districts namely: Butaleja, Bududa, and Manafwa with a total population of 808,151 and a population density of 906 persons/ km^2^ of which 203,262(25.2%) are school going. Of the school going, 40,442 (20%) are adolescents aged between 13 and 18 years [32]. Manafwa watershed has approximately 40 secondary schools.

### Study population and eligibility screening

The target population for this study was school-going adolescents aged between 10 and 18 years. Only adolescents who were below 19 years and were in secondary school, were considered eligible for participation. We enrolled participants from government-aided secondary schools within the Manafwa watershed area who provided written informed assent. For participants under 18 years old, written permission from their parent or guardian was obtained in addition to informed consent. School-going adolescents aged 18 years provided informed consent directly We excluded adolescents who missed school at the time of data collection, and adolescents who were sick and unable to participate in the study.

### Sample size estimation

Sample size calculation was done using the Kish Leslie formula for single proportion as indicated below [33]. A prevalence of 31.3% for depression and anxiety among children and adolescents during pandemic [34]. We adjusted for clustering using a design effect of 2 and also adjusted for 15% non-response.

$$N\; = \;{{{Z^2}{}_{\alpha /2}p(1 - p)} \over {{d^2}}}\;*\;DE$$

P = prevalence of 31.3% for depression and anxiety among children and adolescents during pandemic [35].

Z = Standard normal value corresponding to 95% level of confidence (1.96).

DE = Design effect of 2.

d = tolerable sampling error 0.05.

This gave *N* = 662.

We then adjusted for 15% non-response. A sample size of 762 was then obtained.

Therefore *N* = 762.

### Sampling procedures and data collection

We used proportionate stratified random sampling to select the number of adolescents to be recruited per district. The proportions were based on the total number of students in all the schools per district. A total of 250, 256, and 256 was recruited from Manafwa, Bududa, and Butalejja respectively. Four secondary schools were selected per district basing on population and participants were then selected randomly. A sampling frame for each school was generated by registering the students in the classes on the day of data collection and random sampling was done for the participants. Unique numbers were assigned to all adolescents from each class and then used a random number generator plus app to select adolescents to be included in the study. Eligible participants underwent informed consent process and questionnaires were administered. For participants, less than 18 years, the parent/guardian permission was given to the children to take to the parents and brought back. The paper-based questionnaires were administered upon consent of both parties. The questionnaire was self-administered by the adolescents. Data was then checked for completeness and later entered using kobo collect.

### Study measures and tools

A pretested questionnaire was used to collect data from the participants. The questionnaire included socio-demographic information (age, sex, orphanhood, household head, class, nature of family, number of school-going children in home, number of adults in home and number children in home) and copying strategies. Stages of adolescence were divided into early (10–14 years), middle (15–17) and late (18–19) [35].

#### KidCope tool

KidCope tool was used to evaluate adolescents’ coping strategies [36]. KidCope enables adolescents to analyze ten coping strategies (distraction, social withdrawal, self-criticism, blaming others, emotional regulation, problem-solving, cognitive restructuring, wishful thinking, social support, and resignation) [37]. The KidCope older version for 13–17 years is a Likert-type scale that is scored for questions assessing the frequency of use for each coping strategy on a 4-point, not at all to almost all the time and the effectiveness of the coping strategy or how beneficial it proved to be; on a 5-point not at all to very much. The KidCope tool has an internal consistency (alpha) estimate ranging from 0.30 to 0.61 [36]. This tool is self-administered and has been utilized in different cultures and countries including African countries like Sudan and Uganda [38] for measuring coping of children and adolescents. In this study, the internal consistency or reliability of the tool measured by the Cronbach Alpha was 0.5651 which falls between the estimated range for the KidCope tool (0.3–0.61) though a bit low according to the rule of thumb [36].

### Data analysis

Data were analyzed using in Stata version 14.0 (Stata Corp LLC, College Station, Texas, USA). Categorical variables were summarized using frequencies and percentage. The social demographic characteristics were stratified by sex of the participants. Chi square test was used to analyze the differences between male and female regarding the social demographic characteristics.

Independent Samples t-test was used to determine the difference of the mean scores of the coping strategies between males and females. One-Way Variance of Analysis for Independent Samples (ANOVA) was used for comparing the mean scores acquired from the scales among the stage of adolescence (early (< 15), middle (15–17) and late (> 17)). One-way analysis of variance (ANOVA) was followed by Bonferroni post hoc test, if F-value was found to be significant (P-value < 0.01). The Bartlett’s test for equal variances was used to test for homogeneity if the assumptions of normality was fulfilling. To identify categories of copying strategies based on patterns of correlation from strategies in the Kid cope, factor analysis was done using scree plot and apriori criteria [39]. The apriori criterion was based on the number of factors used in previous studies and the Scree plot was a plot of eigen values (amount of variance accounted for by the factors) [39]. For this study we adopted a three-factor model as all items were loading on all factors and no items were cross-loading. The scree plot criterion was also used and the number of factors were determined at the point of inflexion of the curve. Composite scores were obtained for the three factors, and they were categorized as less than one (not using a particular coping strategy category) and greater or equal to one (using a particular coping strategy category). The three factors obtained were used for further analysis to evaluate the indifferences of the categories of coping strategies among stages of adolescence and gender.

## Results

A total of 762 individuals participated in the study. More than half (67.9%) of the participants in the middle adolescent stage (15-17yrs) were female. On average, males were older than females i.e., mean age of 17.0 vs. 16.4 respectively, p-value = 0.008. For details see Table 1.

**Table 1:** Distribution of the social demographic characteristics across sex of 762 school-going children in Manafwa watershed area

Variable	Category	Female *n* (%)	Male *n* (%)	*P*-value
		446 (67.9)	316 (32.1)	
Age (mean, SD)		16.4 (1.3)	17.0 (1.2)	**0.008**
Stage of adolescence (13–18 years)	Early (< 15)	38 (8.5)	18 (5.7)	0.240
	Middle (15–17)	303 (67.9)	213 (67.4)	
	Late (> 17)	105 (23.5)	85 (26.9)	
Nature of family	Monogamous	258 (57.9)	172 (54.4)	0.549
	Polygamous	118 (26.5)	86 (27.2)	
	Single parent	70 (15.7)	58 (18.4)	
Household head	Child	1 (0.2)	5 (1.6)	0.090
	Father	369 (82.7)	252 (79.8)	
	Mother	76 (17.0)	59 (18.7)	
Orphanhood	Not orphan	384 (86.1)	269 (85.1)	0.706
	Orphan	62 (13.9)	47 (14.9)	
District	Bududa	154 (34.5)	102 (32.3)	0.475
	Butaleja	142 (31.8)	114 (36.1)	
	Manafwa	150 (33.63)	100 (31.7)	
Number of children in household	0–5	162 (36.3)	110 (34.8)	0.668
	6–22	284 (63.7)	206 (65.2)	
Number of adults in household	1–4	185 (41.5)	133 (42.1)	0.867
	4–22	261 (58.5)	183 (57.9)	
Number of school children in household	0–5	219 (49.1)	147 (46.5)	0.482
	6–18	227 (50.9)	169 (53.5)	

### Copying strategies

#### Factor analysis for the coping strategies

**Fig. 1:**
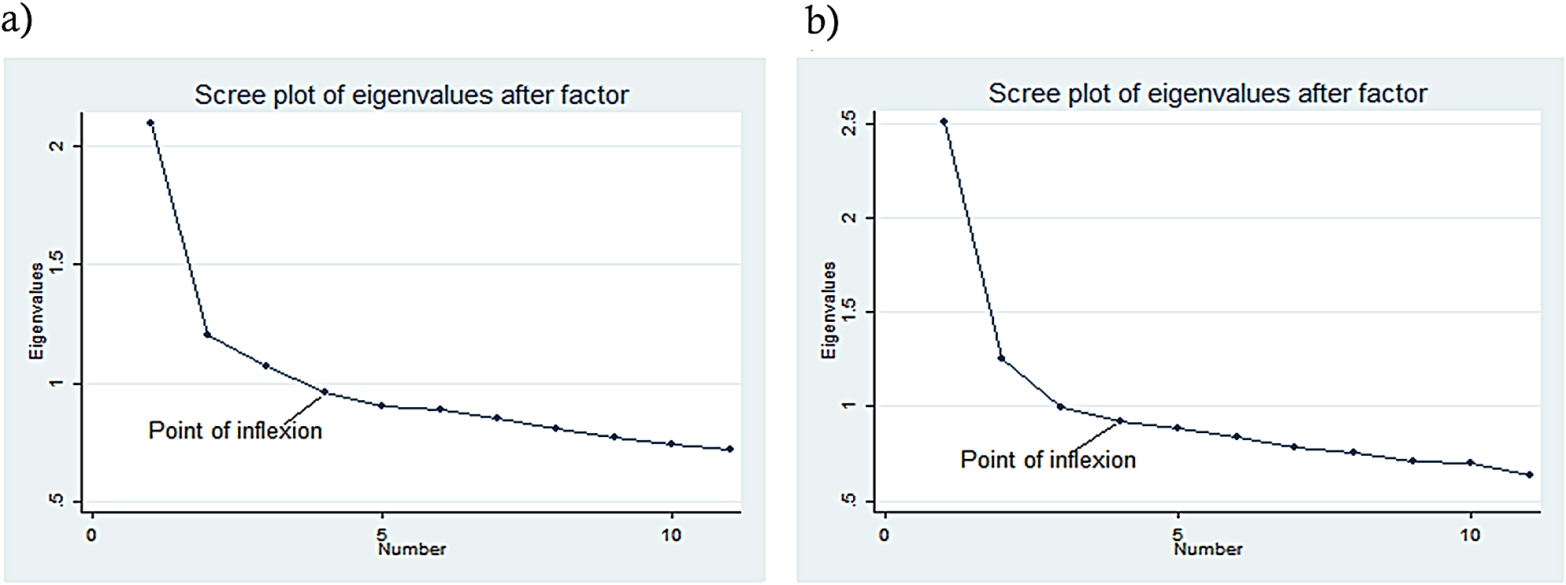
Scree plots of the frequency and efficacy of the coping strategies used by school going adolescents in Manafwa water shed area. (**a**) Frequency (**b**) Efficacy

The scree plot criterion showed that the point of inflexion (indicated by an arrow) was on four factors but due to subjectivity of this criterion we subtracted one from the four hence three factors were to be obtained. For the apriori criterion, which is based on previous studies [40, 41], three factors were used. Therefore, from both criteria (Scree plot and apriori), three factors were extracted for both frequency (how often they used the coping strategy) and efficacy (how helpful the coping strategy was) of the coping strategies. (Fig. 1)

#### Categories of the coping strategies used by school going adolescents in Manafwa watershed area

Three factors explained 39.72% of the variance. (Table 2). The table only shows the variances greater than 0.3 because the small coefficients were suppressed at 0.3, so those less than 0.3 were excluded.

Factor 1 included five items that explained 14.63% of the variance and labelled **adaptive coping** that measured distraction (item 1), cognitive restructuring (item 3), problem-solving (item 6), positive emotional regulation (item 7b), and social support (item 9).

Factor 2 included four items that explained 13.61% of the variance and labelled **avoidant coping** that measured social withdrawal (item 2), Blaming others (item 5), wishful thinking (item 8), resignation (item 10).

Factor 3 included two items that explained 11.47% of the variance and labelled **negative emotion coping** that measured self-criticism (item 4), and negative emotional regulation (item 7a).

**Table 2:** Distribution of correlations or variable loadings among the 3 factors / categories of the coping strategies

Item / Variable			
	Factor 1	Factor 2	Factor 3
(1) I thought about something else; tried to forget it; and/or went and did something like watch TV or play a game to get it off my mind.	**0.3322**	0.3062	
(2) I stayed away from people; kept my feelings to myself; and just handled the situation on my own.		**0.5884**	
(3) I tried to see the good side of things and/or concentrated on something good that could come out of the situation.	**0.6247**		
(4) I realized I brought the problem on myself and blamed myself for causing it.			**0.7515**
(5) I realized that someone else caused the problem and blamed them for making me go through this.		**0.4212**	0.3138
(6) I thought of ways to solve the problem; talked to others to get more facts and information about the problem and/or tried to actually solve the problem.	**0.6857**		
(7a) I talked about how I was feeling; yelled, screamed, or hit something.			**0.6230**
(7b) Tried to calm myself by talking to myself, praying, taking a walk, or just trying to relax.	**0.3677**	0.3521	
(8) I kept thinking and wishing this had never happened; and/or that I could change what had happened.	0.3512	**0.5319**	
(9) Turned to my family, friends, or other adults to help me feel better.	**0.5695**		0.3438
(10) I just accepted the problem because I knew I couldn’t do anything about it.		**0.6490**	
**% of variance**	14.63	13.61	11.47
**Cumulative%**	14.63	28.24	39.72

*Extraction Method*: *Principal Component Analysis*. Rotation method: orthogonal varimax (Kaiser off

### Distribution of individual coping strategies used and perceived effectiveness

Overall, social support and positive emotion regulation strategies under the adaptive coping category were the most frequently used coping strategies (*n* = 712, 93.4%) and (*n* = 708, 92.9%) respectively. These strategies were also perceived as the most effective coping strategies, with a mean of (mean/SD = 3.1 ± 1.2) for social support and (mean/SD = 2.9 ± 1.3 for positive emotional regulation respectively. (Table 3)

**Table 3:** Distribution of the used copying strategies and their perceived effectiveness

Coping Strategy	Used strategy		Efficacy Mean ± SD
	No (*n* (%))	Yes (*n* (%))	
**Adaptive coping**			
Distraction	61 (8.0)	701 (92.0)	2.3 ± 1.4
Cognitive restructuring	55 (7.2)	706 (92.8)	2.5 ± 1.3
Problem-solving	60 (7.9)	702 (92.1)	2.8 ± 1.3
Positive emotional regulation	54 (7.1)	708 (92.9)	2.9 ± 1.3
Social support	50 (6.6)	712 (93.4)	3.1 ± 1.2
**Avoidant coping**			
Social withdrawal	197 (25.9)	565 (74.2)	2.0 ± 1.5
Blaming others	155 (20.3)	607 (79.7)	1.7 ± 1.5
Wishful thinking	93 (12.2)	669 (87.8)	2.3 ± 1.5
Resignation	251 (32.9)	511 (67.1)	1.9 ± 1.5
**Negative-emotion coping**			
Self-criticism	240 (31.5)	522 (68.5)	1.6 ± 1.5
Negative emotional regulation	199 (26.1)	563 (73.9)	1.8 ± 1.5

### Distribution of the coping strategies across the sex of the participants

Females significantly employed higher resignation as a coping strategy compared to males, (mean of 1.2 vs. 1.0, respectively; *P* = 0.026). (Table 4)

**Table 4:** Distribution of the coping strategies across sex

Coping strategy	Sex		
	Male *n* = 316 Mean ± SD	Female *n* = 446 Mean ± SD	*P*-value
**Adaptive coping**			
Distraction	1.6 ± 0.9	1.5 ± 0.9	0.741
Cognitive restructuring	1.9 ± 0.9	1.8 ± 0.9	0.621
Problem-solving	1.8 ± 1.0	1.8 ± 1.0	0.858
Positive emotional regulation	1.9 ± 1.0	2.0 ± 1.0	0.341
Social support	2.0 ± 1.0	2.0 ± 1.0	0.365
**Avoidant Coping**			
Social withdrawal	1.2 ± 1.0	1.3 ± 1.0	0.277
Blaming others	1.3 ± 1.0	1.3 ± 1.0	0.684
Wishful thinking	1.7 ± 1.0	1.7 ± 1.0	0.643
Resignation	1.0 ± 1.0	1.2 ± 1.0	**0.026**
**Negative-emotion coping**			
Self-criticism	1.1 ± 0.9	1.0 ± 1.0	0.189
Negative emotional regulation	1.2 ± 1.0	1.2 ± 1.0	0.824

### Distribution of coping strategies across the stage of adolescence

Adolescents in the three age groups that used the Distraction coping method showed a statistically significant mean difference (P-value = 0.010). (Table 5).

Multiple comparisons were performed using the Bonferroni post hoc test on the Distraction coping strategy. This revealed that adolescents in the early stage used more distraction than those in the middle adolescence stage (mean difference = 0.36, *p* = 0.013). (Table 6)

**Table 5:** Distribution of strategies frequency across the adolescent stage

Coping strategy	Age categories (Stage of adolescence)				
	Early *n* = 153 Mean ± SD	Middle *n* = 609 Mean ± SD	Late *n* = 609 Mean ± SD	Test of significance	
				F-value	*P*-value
**Adaptive coping**					
Distraction	1.3 ± 0.9	1.6 ± 0.9	1.5 ± 0.9	4.59	**0.010**
cognitive restructuring	1.8 ± 1.0	1.8 ± 0.9	1.9 ± 1.0	0.20	0.818
Problem-solving	1.8 ± 1.0	1.8 ± 1.0	1.9 ± 0.9	0.17	0.845
Positive-emotional regulation	2.0 ± 1.1	2.0 ± 1.0	2.0 ± 1.0	0.15	0.861
Social support	2.1 ± 1.1	2.0 ± 1.0	1.9 ± 1.0	0.53	0.589
**Avoidant coping**					
Social withdrawal	1.1 ± 0.9	1.3 ± 1.0	1.3 ± 1.1	1.15	0.318
Blaming others	1.5 ± 1.0	1.3 ± 1.0	1.4 ± 1.0	1.17	0.310
Wishful thinking	1.8 ± 1.0	1.7 ± 1.0	1.7 ± 1.0	0.84	0.433
Resignation	1.3 ± 1.0	1.1 ± 1.0	1.1 ± 1.0	1.55	0.214
**Negative-emotion coping**					
Self-criticism	1.1 ± 0.9	1.1 ± 1.0	1.0 ± 1.0	1.58	0.208
Negative-emotional regulation	1.3 ± 1.1	1.2 ± 1.0	1.2 ± 1.0	0.14	0.869

**Table 6:** Post hoc analysis showing comparisons of the Distraction coping by age category of the participants

Coping strategy	Comparisons	Difference in means	*P*-value
Distraction	Early vs. Middle	0.36	**0.013**
	Early vs. Late	0.25	0.189
	Middle vs. Late	-0.11	0.465

### Distribution of the categories of coping strategies by stage of adolescence and sex of the participants

**Fig. 2:**
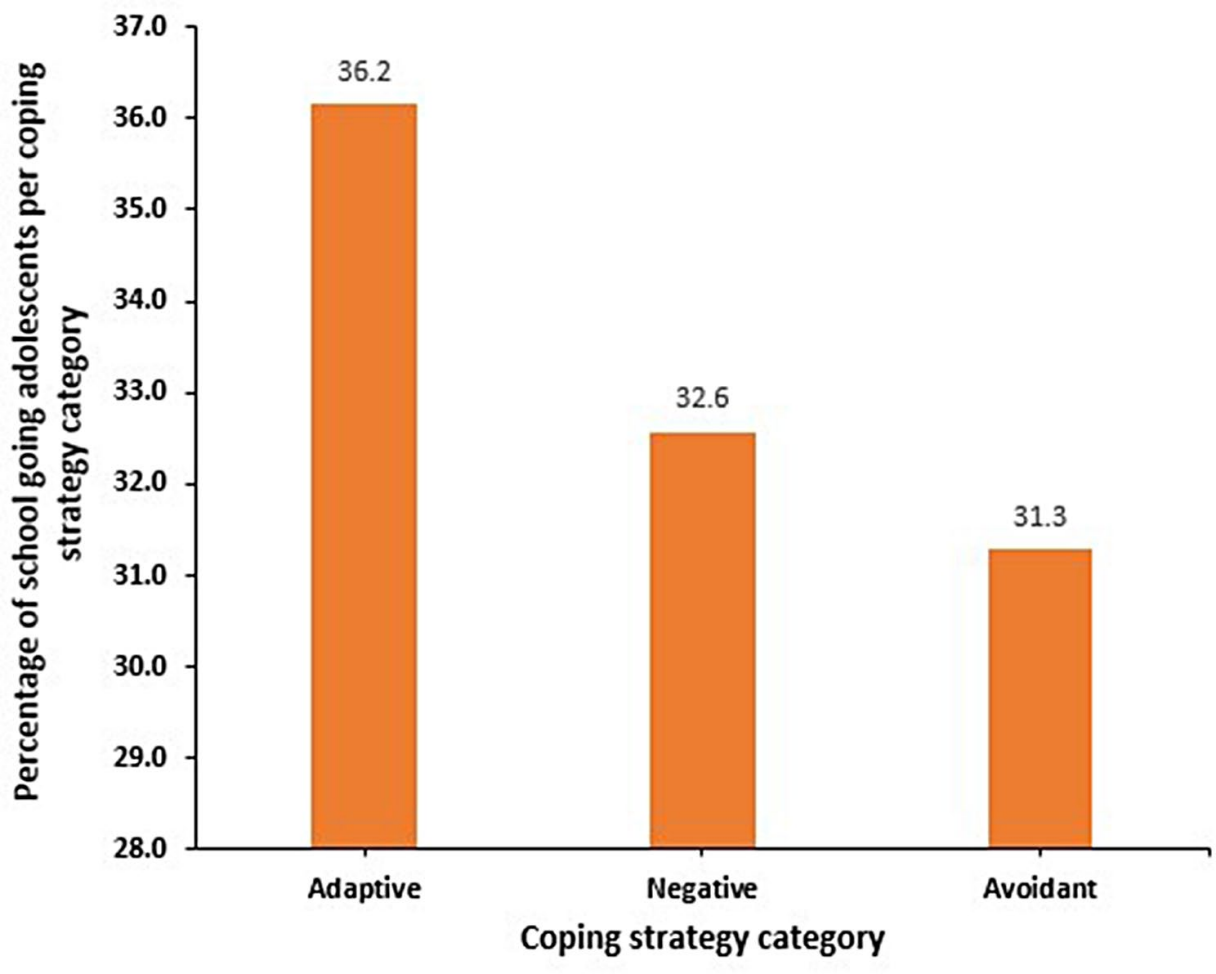
Categories of the copying strategies

Majority (36.2%) of the participants employed adaptive coping followed by negative-emotion coping (32.6%) and avoidant coping (31.3%). (Fig. 2)

There was a statistically significant higher proportion of middle age adolescents that used negative emotion coping i.e., middle age (69.3%) vs. late adolescence (18.9%) vs. early adolescent stage (11.8%), P-value = 0.040. There was no statistically significant difference in categorized copying strategies employed across sex of the participants. (Table 7)

**Table 7:** Comparison of the 3 coping strategy factors by stage of adolescence and gender of the participants

Variable	Category	Adaptive coping *n* (%)			Avoidant coping *n* (%)			Negative-emotion coping *n* (%)		
		No 620 (81.3)	Yes 141 (18.5)	*p* -value	No 639 (83.9)	Yes 122 (16.0)	*p*-value	No 634 (83.2)	Yes 127 (16.7)	*p*-value
Stage	Early (< 15)	42 (6.8)	14 (9.9)	0.426	49 (7.7)	7 (5.7)	0.679	41 (6.5)	15 (11.8)	**0.040**
	Midde (15–17)	423 (68.2)	92 (65.3)		433 (67.8)	82 (67.2)		427 (67.4)	88 (69.3)	
	Late (> 17)	155 (25.0)	35 (24.8)		157 (24.6)	33 (27.1)		166 (26.2)	24 (18.9)	
Gender	Female	366 (59.0)	79 (56.0)	0.514	370 (57.9)	75 (61.5)	0.463	366 (57.7)	79 (62.2)	0.350
	Male	254 (41.0)	62 (44.0)		269 (42.1)	47 (38.5)		268 (42.3)	48 (37.8)	

## Discussion

This study aimed to assess the coping strategies employed by the school going children during the COVID19 pandemic in the Manafwa water shed area, Uganda. Three main categories of coping strategies were adopted by school-going adolescents i.e., adaptive, avoidant and negative-emotion coping strategies. Majority of the participants employed adaptive coping and avoidant coping was least employed. Of the adaptive copying strategies, positive emotional regulation and social support were most used and were considered most effective. In this study, resignation as a copying strategy was used more in female adolescents as compared to their male counter pants and this was statistically significant. A higher proportion of middle-aged adolescents employed negative-emotion regulation. However, those in early adolescence used distraction to cope more than those in middle adolescence.

Engaging in adaptive coping strategies, whether through problem-focused coping or positive emotion-focused coping, is consistently linked to positive outcomes and enhanced psychological well-being [24, 26, 28]. Adaptive problem-focused coping involves actively addressing challenges, fostering a sense of control and accomplishment, leading to improved mental well-being [24, 26, 28]. Similarly, adaptive positive emotion-focused coping, including strategies like reappraisal and relaxation techniques, contributes to emotional regulation and resilience [24, 26, 28]. Contrary, employing negative emotion-focused coping during the COVID-19 quarantine is associated with poorer psychological health, such as increased anxiety and depression and [23]. Comparably, avoidant coping involves attempting to create distance from stressors or avoiding direct confrontation, often downplaying the importance of the stressor [23]. While these strategies may provide momentary relief, they are generally less effective in addressing the underlying causes of stress, potentially leading to prolonged emotional distress [23].

The high prevalence of adaptive coping strategies (which involves distraction, problem-solving, seeking social support, and maintaining a positive outlook) in this study’s findings suggests a degree of resilience among school-going adolescents in Uganda. This finding also highlights the significance of social support structures within the African communities [42]. This may be indicative of a positive trend in terms of mental health resilience among school-going adolescents in Uganda during the pandemic. These findings are consistent with those from a study done in Ghana [43] among adolescents living with HIV.

More to note, in this study, the most effective strategies were the most frequently used, unlike findings a study among adolescents with spinal cord injury in United States of America [44]. This may be due to differences in challenges across the study populations and differences in study settings with commonly utilized strategies like seeking social support, in this study, being culturally perceived as more effective in the African context [42]. Furthermore, this study was carried out in an area that has been identified as one of Uganda’s most vulnerable regions because of the area’s ongoing exposure to the consequences of climate change, including landslides [45]. Changes in society brought about by the pandemic might have reinforced the copying that this group already possessed [45].

Findings from this study revealed that female adolescents used resignation as a coping strategy as compared to their male counterparts. These findings echo to those in a study conducted in the Democratic Republic of Congo (DRC) that aimed at evaluating age and gender differences in potentially traumatic event exposure and coping strategies [46]. That study in DRC revealed that resignation was mostly utilized by girls as compared to boys to reduce their internalizing and externalizing problems [46]. It is possible that societal expectations and traditional gender roles, which dictate that males should handle problems while females should wait may cause female adolescents to use resignation as a coping mechanism to accept their roles [47, 48]. It is also possible that during pandemic, females predominantly found themselves confined to their homes, taking care of younger siblings, in contrast to males. Consequently, they tended to resort to a resignation strategy.

The study finding that adolescents in the early stage employed more of distraction as a coping strategy is likely because during early adolescence, the brain under goes synaptic pruning and strengthens existing neural connections [49, 50]. Therefore it may not be entirely prepared to tackle difficult or unfamiliar situations, such those presented by unexpected occurrences like the COVID-19 pandemic [49, 51]. Adolescents may use distraction as a coping mechanism for the cognitive difficulties of changing course in unexpected situations. These findings contradict those from previous studies among adolescents in Australia and Germany [41, 52, 53], who demonstrated lower levels of adaptive strategies like distraction. This is likely because the studies conducted in Australia and Germany assessed for coping with common stressors [45–47], while the stressor in this study was uncommon.

The finding that majority of the participants in the middle adolescent stage used more of negative emotion coping as compared to other stages aligns with findings from studies [54, 55]. While there is a great deal of identity exploration during the middle adolescent stage [56], the pandemic’s uncertainty and the hormonal changes that occur during adolescence can negatively impact mood regulation and making positive copying mechanisms more challenging [57]. In addition, those in middle adolescence turn more and more to their friends for peer interactions, social validation, and support—aspects that were prohibited during the pandemic [14, 58, 59]. This possibly caused them to resort to negative emotion management strategies, like self-criticism, to cope with the challenging situations elicit. Furthermore, middle adolescence is a period of ongoing cognitive growth, and as a result, their emotional regulation skills are still developing, which makes them more likely to rely on ineffective strategies [60]. Moreover, some middle-aged teens may experiment with risky behaviors in trying to explore their autonomy and identity under trying conditions, which can lead to negative emotion coping [61]. We therefore recommend some supportive mechanisms and interventions like Cognitive Behavioral Therapy and psychoeducation in these schools to regulate their negative emotion coping and navigating various challenges they may encounter.

## Strengths and limitations of the study

The biggest strength of this study is that the findings from this study are timely as they will help policy makers to set up better interventions or mechanisms that can be used by adolescents to avoid distress that could be brought by any future calamities like pandemics.

Some limitations should be taken into account when interpreting the findings of this study. Firstly, because there were more females than males in the study hence selection bias was likely to have occurred. Secondly, the study was conducted retrospectively resulting to be recall bias due to seeking answers about events that happened during the COVID-19 pandemic. We reduced this by limiting the number of questions being asked. The use of a descriptive cross-sectional study was a limitation as we were not able to assess the casual effect of different factors on the coping strategies used by the school going adolescents. Additionally, the KidCope tool’s low internal consistency, as indicated by its Cronbach alpha of 0.5651, could have affected the results because the questions were not consistently related. Additionally, this study did not capture the adolescents who never returned to school after COVID. This would in essence create a selection bias. However, we also assessed the proportion of those who were in school before and after the pandemic and only 15.5% of the school going children never returned to school. Therefore, there was a low selection bias. The strength of this study is that it had a large sample size reducing likelihood of random error. The study was conducted in about twelve schools in the Manafwa watershed area from three districts increasing generalizability of the findings.

### Conclusion

The majority of school-going adolescents demonstrated a preference for adaptive coping strategies, with social support and positive emotional regulation being the most frequently used and perceived as the most effective; suggesting a degree of resilience among the school-going adolescents in Uganda. Females were found to use resignation more frequently than males, suggesting potential challenges in adjustment and a higher level of emotional sensitivity among female adolescents. Additionally, middle-aged adolescents were more inclined towards negative emotion coping, possibly due to the challenges of identity exploration, hormonal changes, and limited peer interactions during the pandemic.

### Recommendations

We recommend the ministry of education to incorporate mental health education into the school curriculum, covering topics such as stress management, emotional well-being, and positive coping strategies. These lessons can help students, the middle adolescents learn how to cope with changing environments. The ministry should also encourage teachers, create gender-neutral environments within schools that encourage both male and female students express their emotions and seek support without feeling constrained by traditional gender norms. We recommend training of health providers especially mental health service providers in identification of negative copying strategies. We also recommend that these health providers find creative ways to provide mental health support and services for adolescents on how best to cope with challenging circumstances. We recommend further research in other parts of the country to see whether the age and gender differences also had an effect on school going children while coping with the changes that came as a result of the pandemic. We recommend future analytical prospective cohort studies to be conducted to help assess the causality of the coping strategies among school going adolescents and mixed methods studies to triangulate the results.

## Data Availability

The data supporting the research findings is available from the corresponding author (CB) on reasonable request.

## Abbreviations

ANOVA: Analysis of variance
NAS: National Academic of Sciences
SBS: Social and Behavioral Sciences
SD: Standard Deviation
UNCST: Uganda National Council for Science and Technology
USAID: United States Agency for International Development
